# Supplementary figures and images for: Identification of differential hypothalamic DNA methylation and gene expression associated with sexual partner preferences in rams
Source: PLoS One. 2022 May 12;17(5):e0263319. doi: 10.1371/journal.pone.0263319 (PMC9098078; doi:10.1371/journal.pone.0263319)

Sequence content across all bases FOR1

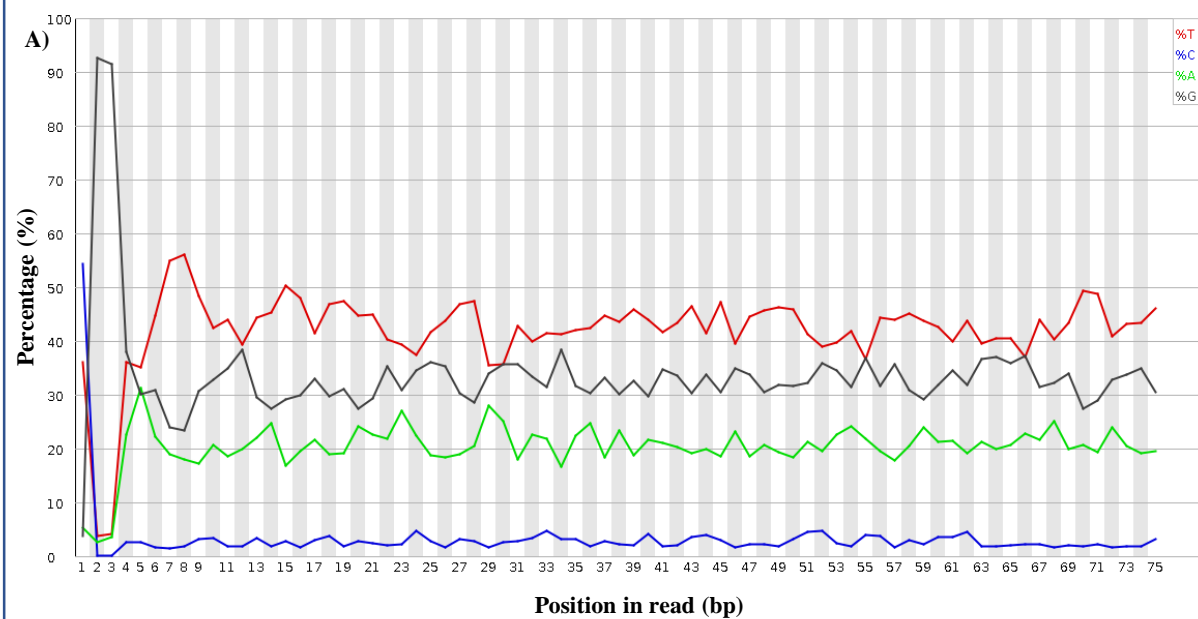

Sequence content across all bases FOR2

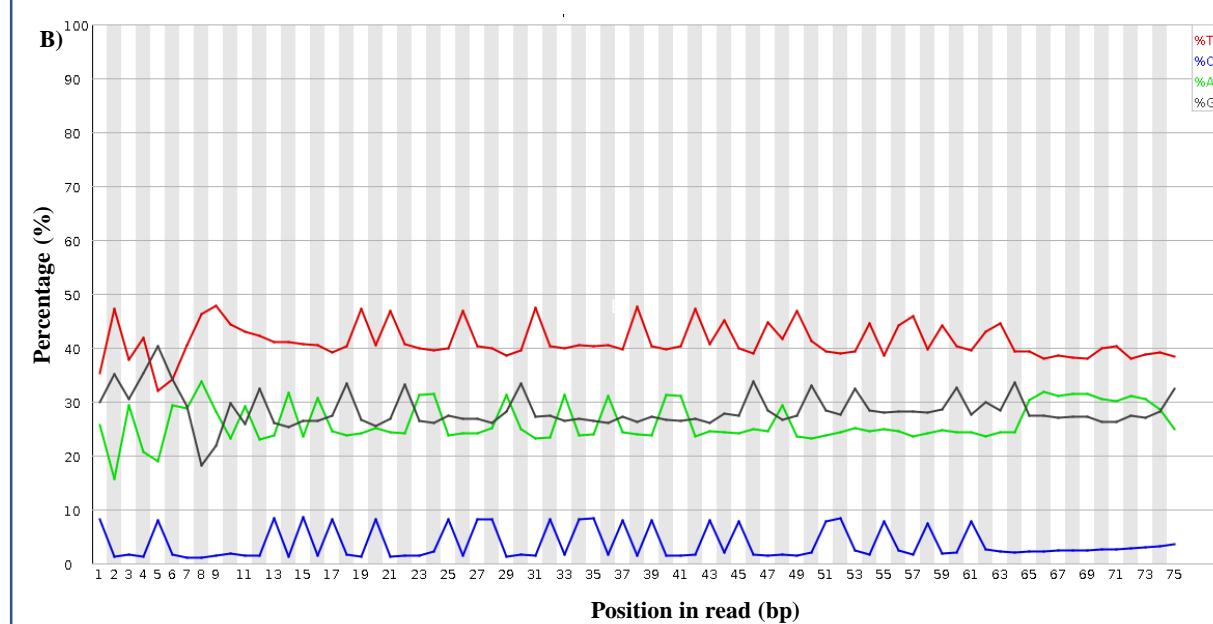

Supplement: S1 Fig — A) Sequence content across all bases for FOR1: Graph showing the representation of the nucleotides across all base position in sample FOR1. Percentage of C (marked in blue) is highest in the first base followed by G (in black) in the next two positions. Similar pattern was observed in all other samples, excepting FOR2. B) Sequence content across all bases for FOR2: Graph showing the representation of the nucleotides across all base position in sample FOR2. Percentage of T (marked in red) is highest in the first three base. Color code Thymine (T) = red, Adenosine = Green, Cytosine = Dark Blue, Guanine = Black. (PDF) [file pone.0263319.s001.pdf]

A)

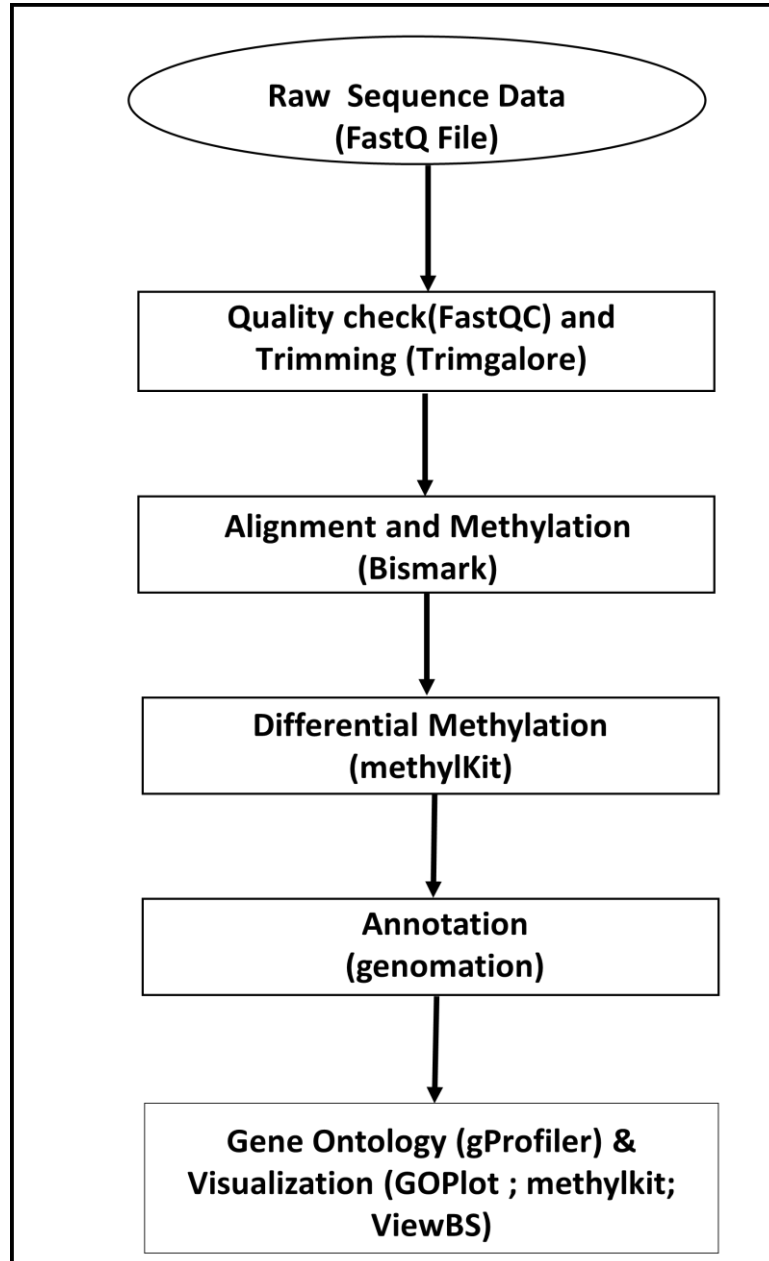

B)

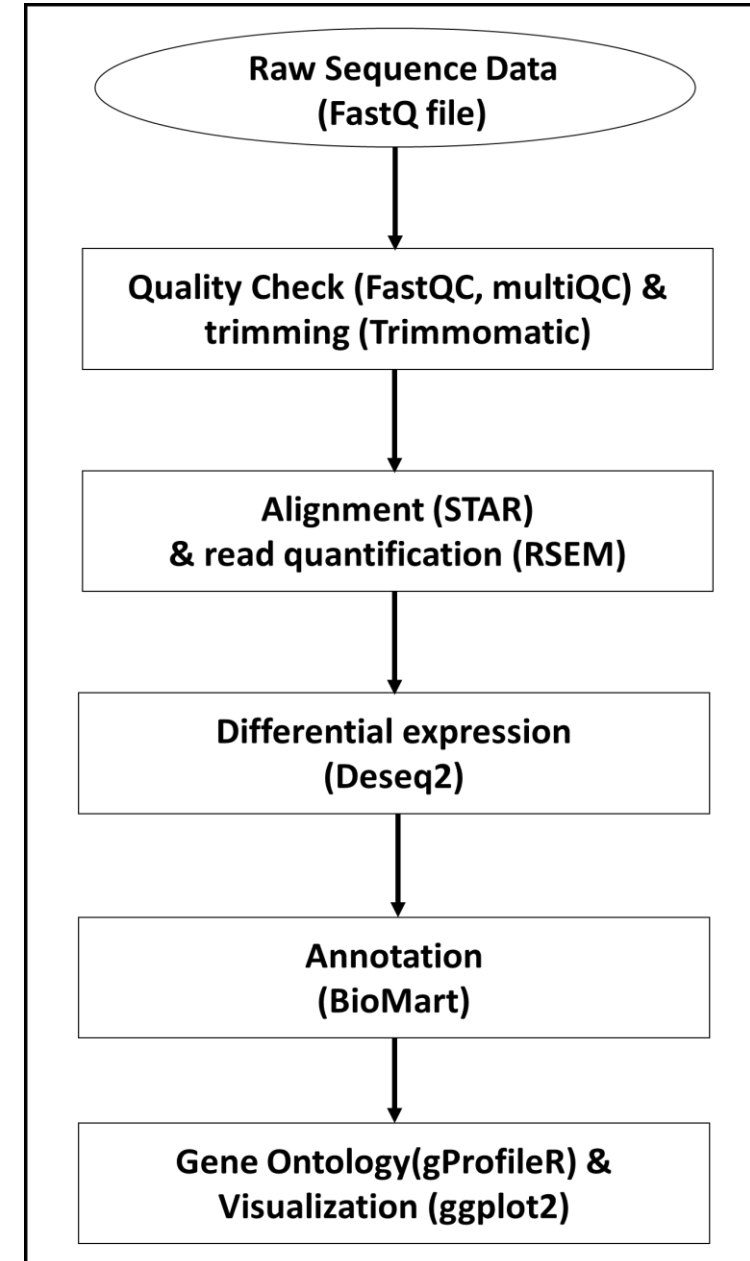

Supplement: S2 Fig — Quality Check using fastqc and trimgalore was used to trim reads less than 20 Phred score. Alignment and methylation count was calculated using Bismark, followed by methylKit to estimate the differentially methylated regions (DMRs) and differentially methylated cytosines (DMCs). Methylation fold change greater than 10; and q value < 0.01 was used for determining the most significant Genes. Annotation of the DMR and DMC was done using genomation. Functional annotation was performed using gProfiler functional annotation tool; followed by visualization using ViewBS for heatmaps, methylKit for dendrogram and distribution of genomic regions for DMRs and GOPlot for gene ontology visualization. (PDF) [file pone.0263319.s002.pdf]

A)

FastQC: Sequence Counts

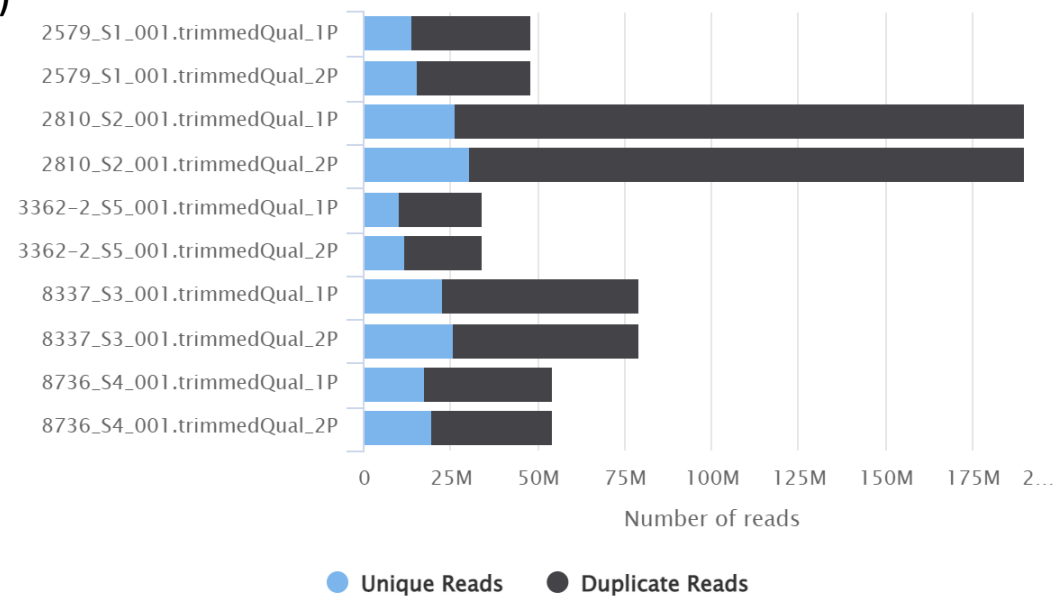

Created with MultiQC

B)

STAR: Alignment Scores

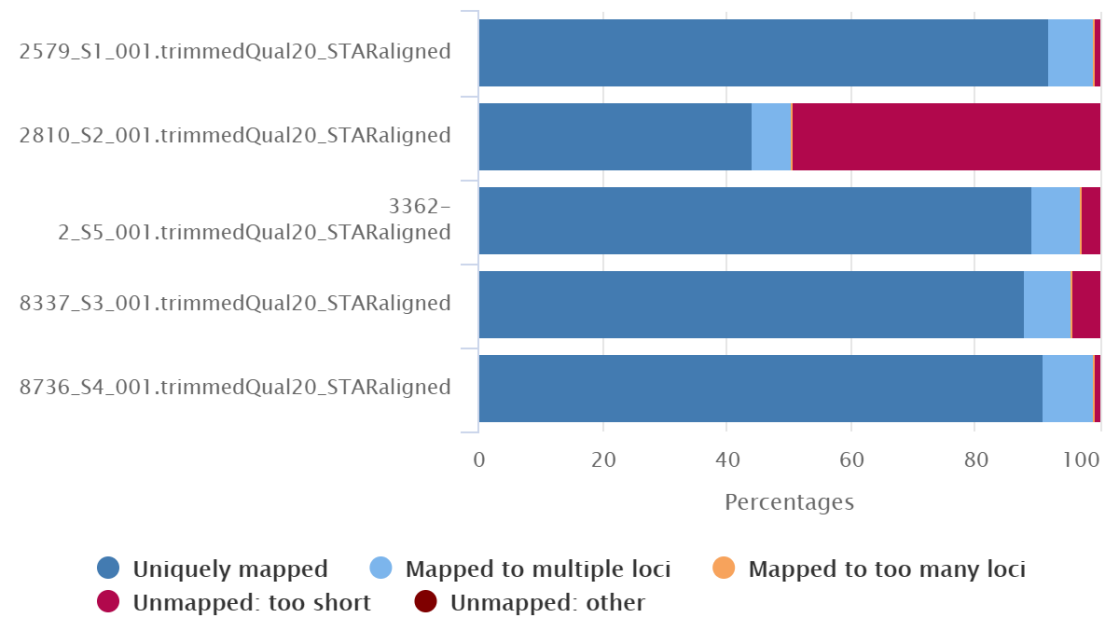

Created with MultiQC

Supplement: S3 Fig — A) Raw read counts from fastq: The plot of the sequence counts shows that for the trimmed sample 2810, the number of reads is greater than 175 million reads, whereas for the other samples has 30 to 75 million reads. We can also observe that the number of duplicate reads (black) in this sample is also greater than the other samples. C) Align read counts from STAR: Aligned read counts from STAR show that sample 2810 has more unmapped reads (red), and least uniquely mapped reads (dark blue) than any of the other samples. (PDF) [file pone.0263319.s003.pdf]

## CpG Hierarchical Clustering

A)

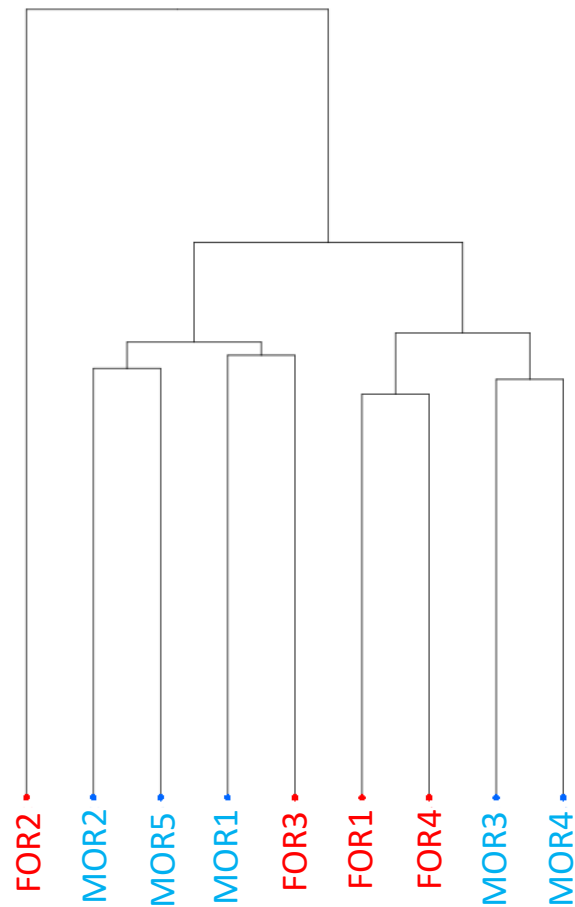

## CHG Hierarchical Clustering

B)

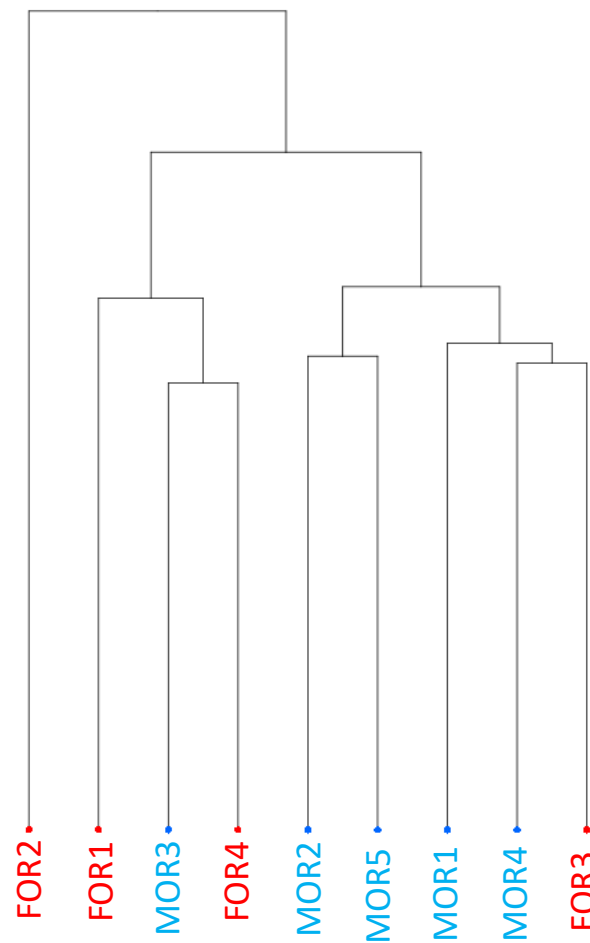

## CHH Hierarchical Clustering

C)

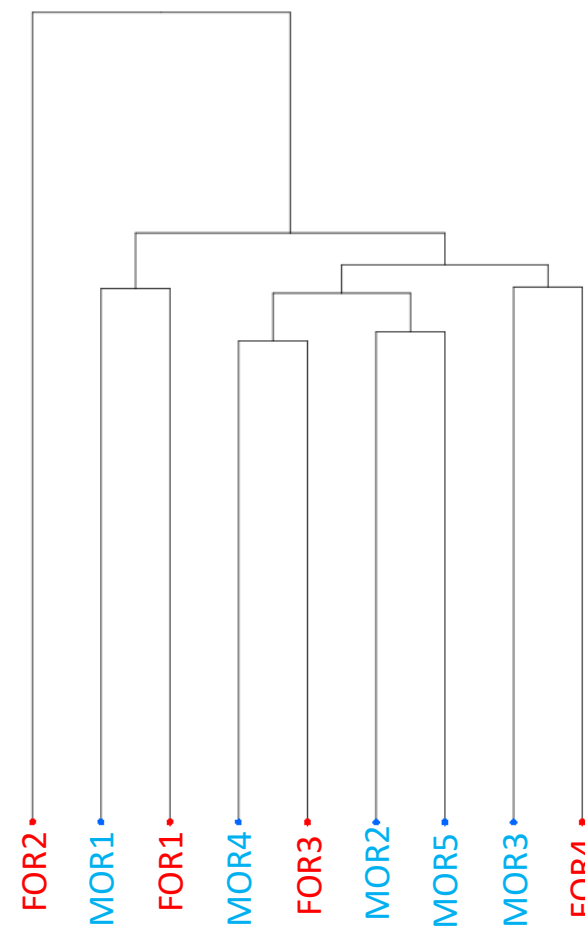

Supplement: S4 Fig — A) CpG hierarchical Clustering: Hierarchical clustering of the methylation pattern of replicates of all the samples, in CpG context) CHG hierarchical Clustering: Hierarchical clustering of the methylation pattern of replicates of all the samples, in CHG context. C) CpG hierarchical Clustering: Hierarchical clustering of the methylation pattern of replicates of all the samples, in CHH context. (PDF) [file pone.0263319.s004.pdf]

## Chromosome X

### CG Context

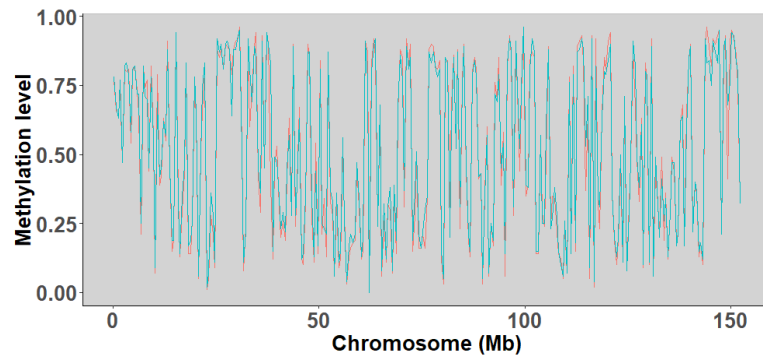

### CHG Context

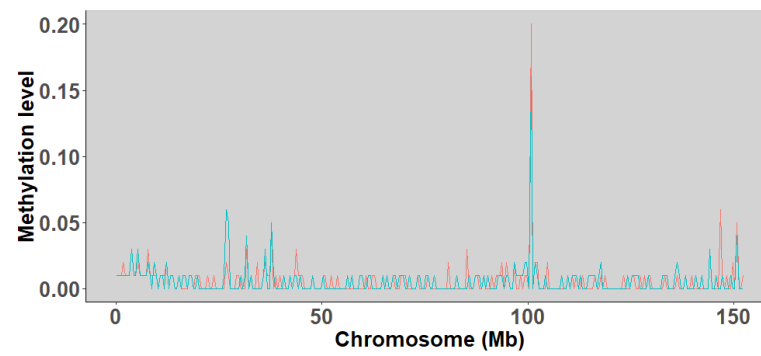

### CHH Context

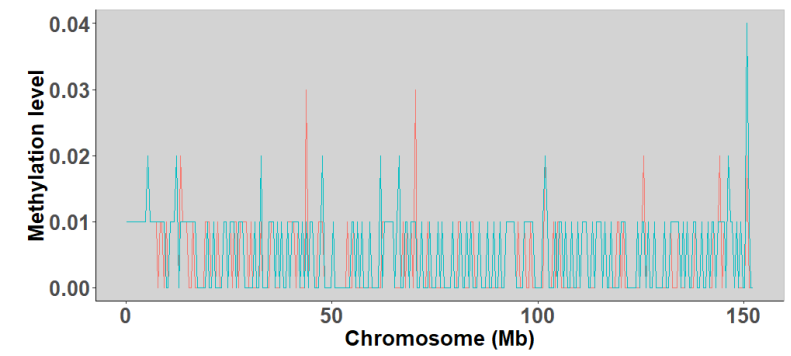

— MOR — FOR

Supplement: S18 Fig — Average methylation levels of the different context between the 2 different samples MOR (red) and FOR (blue). Y-axis average methylation levels, x-axis chromosome coordinates in mega base (Mb). (PDF) [file pone.0263319.s018.pdf]
