# Supplementary material for: Identification of differential hypothalamic DNA methylation and gene expression associated with sexual partner preferences in rams
Source: PLoS One. 2022 May 12;17(5):e0263319. doi: 10.1371/journal.pone.0263319 (PMC9098078; doi:10.1371/journal.pone.0263319)
Supplement: S15 Fig — Average methylation levels of the different context between the 2 different samples MOR (red) and FOR (blue). Y-axis average methylation levels, x-axis chromosome coordinates in mega base (Mb). (PDF) [file pone.0263319.s015.pdf]

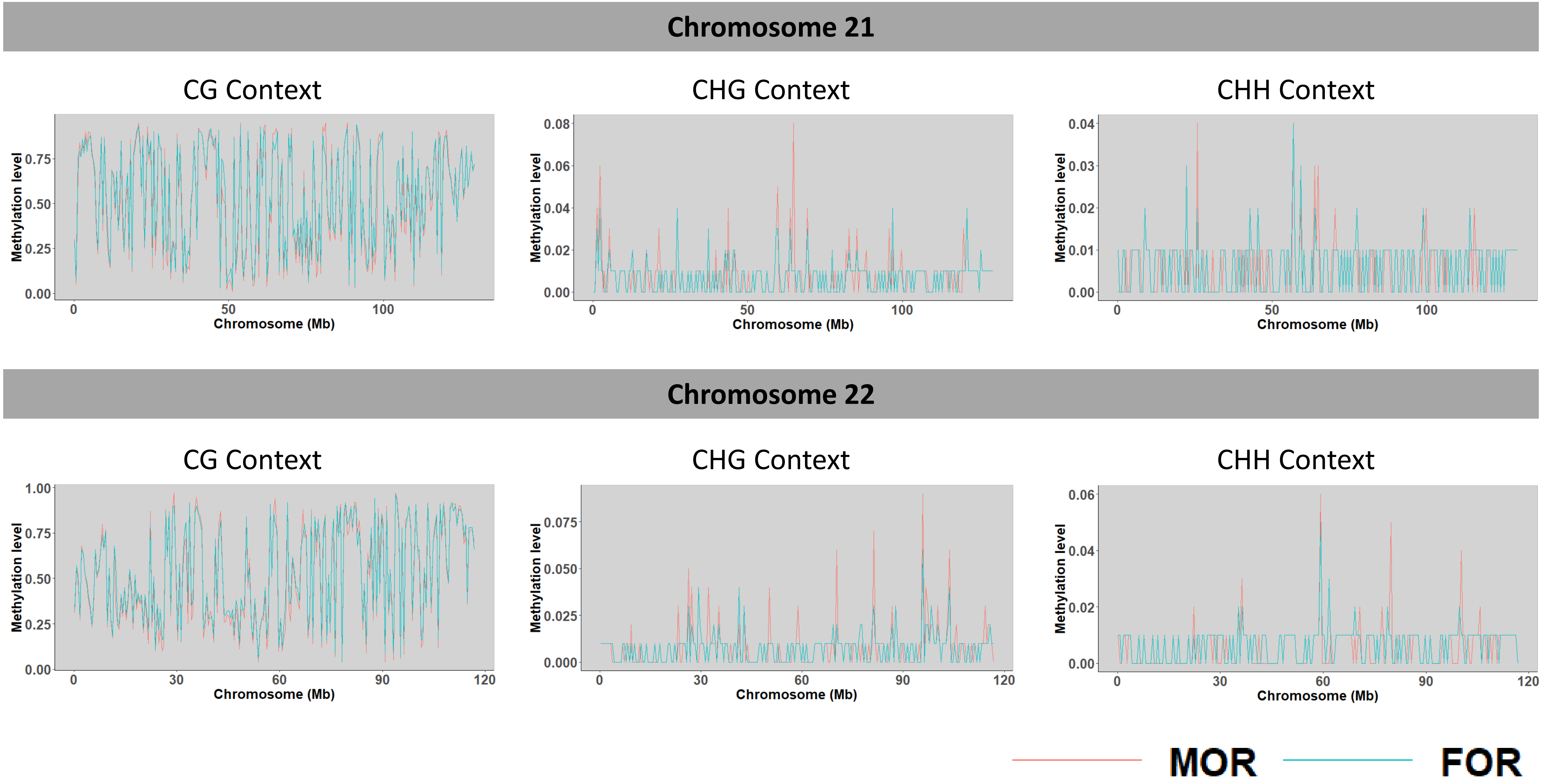

Chromosome 21
CG Context
CHG Context
CHH Context
Methylation level
Chromosome (Mb)
Chromosome 22
CG Context
CHG Context
CHH Context
Methylation level
Chromosome (Mb)
MOR
FOR
