# Supplementary material for: Identification of differential hypothalamic DNA methylation and gene expression associated with sexual partner preferences in rams
Source: PLoS One. 2022 May 12;17(5):e0263319. doi: 10.1371/journal.pone.0263319 (PMC9098078; doi:10.1371/journal.pone.0263319)
Supplement: S17 Fig — Average methylation levels of the different context between the 2 different samples MOR (red) and FOR (blue). Y-axis average methylation levels, x-axis chromosome coordinates in mega base (Mb). (PDF) [file pone.0263319.s017.pdf]

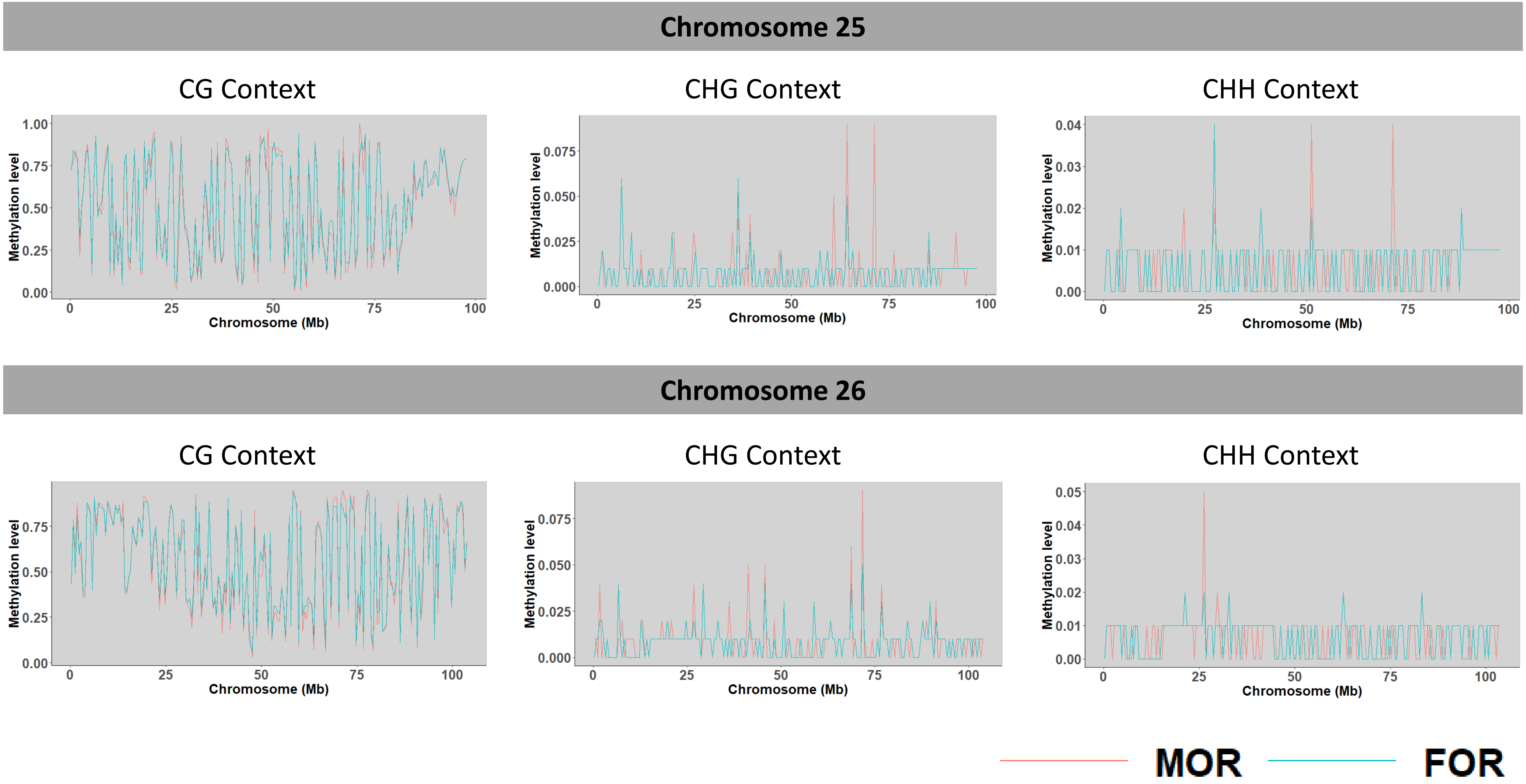
Chromosome 25
CG Context
CHG Context
CHH Context
Methylation level
Chromosome (Mb)
0.00
0.25
0.50
0.75
1.00
0.000
0.025
0.050
0.075
0.00
0.01
0.02
0.03
0.04
0
25
50
75
100
Chromosome 26
CG Context
CHG Context
CHH Context
Methylation level
Chromosome (Mb)
0.00
0.25
0.50
0.75
0.000
0.025
0.050
0.075
0.00
0.01
0.02
0.03
0.04
0.05
0
25
50
75
100
MOR
FOR
