# Supplementary material for: Identification of differential hypothalamic DNA methylation and gene expression associated with sexual partner preferences in rams
Source: PLoS One. 2022 May 12;17(5):e0263319. doi: 10.1371/journal.pone.0263319 (PMC9098078; doi:10.1371/journal.pone.0263319)
Supplement: Attachment — Submitted filename: Plos rebuttal letter 3-8-2022.docx [file pone.0263319.s027.docx]

Response to reviewer

1. We agree that the sample size is a limitation of our study. Although, we commented on this in our original discussion, we added an additional statement to more directly alert the reader to this weakness. See L499-500.
2. As requested, we removed the funding statement from our Acknowledgements.
3. Our updated funding statement should read: “This work was supported by a National Institutes Health grant R01OD011047 and an Oregon Health and Science University School of Medicine Innovation Award both awarded to C.E.R. The funders had no role in study design, data collection and analysis, decision to publish, or preparation of the manuscript. There was no additional external funding received for this study.
4. We provided the GEO accession number (*GSE158287*) in the text (Line 120) and will put a release date on the GEO public access once we get an acceptance.
5. We checked/resaved our figures using PACE
